# Visceral fat changes after distal gastrectomy according to type of reconstruction procedure for gastric cancer

**DOI:** 10.1186/1477-7819-11-146

**Published:** 2013-06-21

**Authors:** Koji Tanaka, Isao Miyashiro, Masahiko Yano, Kentaro Kishi, Masaaki Motoori, Tatsushi Shingai, Shingo Noura, Masayuki Ohue, Hiroaki Ohigashi, Osamu Ishikawa

**Affiliations:** 1 Department of Surgery, Osaka Medical Center for Cancer and Cardiovascular Diseases, 1-3-3 Nakamichi, Higashinari-ku, Osaka 537-8511, Japan

**Keywords:** Gastrectomy, Reconstruction, Visceral fat

## Abstract

**Background:**

Noncancerous causes of death, such as cerebrovascular or cardiac disease, are not rare in patients with gastric cancer who had undergone curative gastrectomy. Metabolic syndrome, characterized by visceral fat accumulation, is a risk factor for cerebrovascular and cardiac diseases. However, there is limited information on the effects of reconstruction procedures on changes in visceral fat after distal gastrectomy. The aim of this study was to analyze the impact of the reconstruction procedure (Roux-en-Y reconstruction (RY) and Billroth I reconstruction (BI)) on changes in visceral fat, as determined using computed tomography.

**Methods:**

The study subjects were 152 patients with gastric cancer who underwent distal gastrectomy with lymphadenectomy between 2002 and 2007. The visceral fat area was measured for one cross-sectional computed tomogram obtained at the level of the umbilicus.

**Results:**

Adjuvant chemotherapy (yes vs. no, *P* = 0.0006), type of reconstruction (BI vs. RY, *P* = 0.0146), field of lymph node dissection (<D2 vs. ≥D2, *P* = 0.0020), omentectomy (yes vs. no, *P* = 0.0003), and pathological stage (1/2 vs. 3/4; *P* = 0.0023) correlated significantly with postoperative visceral fat loss. Multivariate logistic regression analysis identified reconstruction (BI vs. RY; P = 0.0232) and adjuvant chemotherapy (yes vs. no, *P* = 0.0330) as the significant determinants of visceral fat loss after surgery.

**Conclusions:**

Visceral fat loss after RY was larger than that after BI. Further prospective studies are needed to confirm the effects of reconstruction after distal gastrectomy on visceral fat.

## Background

Noncancerous death is a common cause of death in patients with gastric cancer who had undergone curative gastrectomy. This is probably because the 5-year survival rate has improved to about 95%, especially in patients with early gastric cancer treated by radical resection [1]. The causes of noncancerous deaths include cerebrovascular, cardiac, and respiratory diseases [2]. The metabolic syndrome, characterized by visceral fat accumulation, is a risk factor for cerebrovascular and cardiac diseases [3-5]. The Roux-en-Y gastric bypass is a popular procedure used to reduce body weight, and visceral fat, in morbidly obese subjects. Although Roux-en-Y (RY) gastric bypass and Roux-en-Y reconstruction after gastrectomy for gastric cancer are not entirely similar, the selection of reconstruction method could control visceral fat after gastrectomy for gastric cancer.

Several studies have reported poor prognosis of patients with excess post-gastrectomy body weight loss [6,7]. Body weight loss is a serious problem, especially for lean patients. Such weight loss is caused by impaired food intake and malabsorption [8-10] Body weight loss after gastrectomy is thought to be mainly due to loss of body fat (which includes subcutaneous and visceral fat) [11-13]. In this regard, visceral fat change is worthy of remark. However, to our knowledge, there is no information on changes in visceral fat and subcutaneous fat distribution after gastrectomy for different surgical procedures, such as distal gastrectomy with Billroth I (DGBI) reconstruction or distal gastrectomy with Roux-en-Y (DGRY) reconstruction.

In this retrospective study, we first quantified the visceral fat area (VFA) and then determined the impact of different surgical procedures (DGBI and DGRY) on the visceral fat of patients who underwent distal gastrectomy with lymphadenectomy. Clinically, this study serves to provide a basis for appropriate selection of reconstruction method following distal gastrectomy, to provide adequate control of visceral fat volume postoperatively in the long-term.

## Methods

### Patients and surgical procedures

Between January 2002 and April 2007, a total of 152 patients with gastric cancer underwent distal gastrectomy with lymphadenectomy at the Osaka Medical Center for Cancer and Cardiovascular Diseases, Osaka, Japan. They included 106 men and 46 women with a mean age of 62.8 ± 9.8 years (±SD, range: 37 to 85 years; median: 63 years). All patients underwent preoperative assessment, including gastric endoscopy, computed tomography (CT) and laboratory tests. In this study, we compared the CT images taken at least 6 months after surgery with those before surgery.

Roux-en-Y reconstruction was performed when the remnant stomach after resection was too small to allow performance of BI or when the main tumor invaded the pylorus. Lymph node dissection was performed according to the second edition of the *Japanese Classification of Gastric Cancer*[14]. Specifically, patients with T1 and N0 tumors underwent dissection of the perigastric lymph nodes and nodes at the base of the left gastric artery, and along the common hepatic artery, which for simplicity is termed ‘D1+ lymph node dissection’ in this study. Patients with T2 or more advanced tumors and those with N1 or more advanced cancer underwent D2 lymph node dissection, which involves dissection of hepatoduodenal nodes and retropancreatic nodes.

Hospital records were reviewed for age, sex, height, body weight, clinicopathological background data regarding UICC-TNM stage, and surgical procedure. This study was approved by the Human Ethics Review Committee of the Osaka Medical Center for Cancer and Cardiovascular Diseases.

### Quantification of visceral fat area

**Figure 1:**
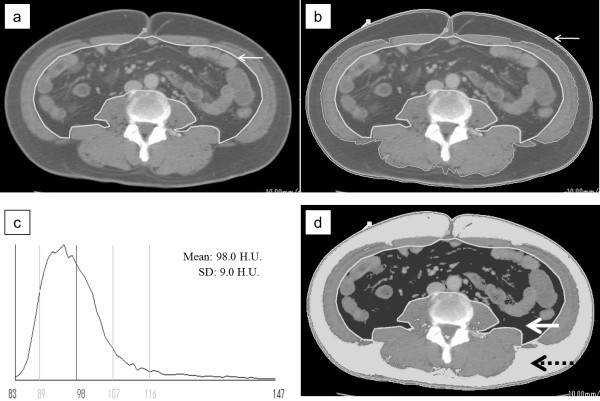
**CT images obtained at the navel level demonstrating the method used to determine abdominal fat distribution.** (**a**) The line (arrowed) outlines the intraperitoneal area. (**b**) The line (arrowed), drawn with a cursor automatically or manually, outlines the subcutaneous fat layer, in which attenuation is measured. (**c**) A histogram of the CT numbers (in Hounsfield units) in the area outlined in b (mean ± 2 SD). (**d**) Measurement of visceral fat tissue (solid arrow). The total fat area is represented by the region outlined by the circumference of the abdominal wall. The visceral fat area was subtracted, and the remainder was regarded as the subcutaneous fat area (dotted arrow).

The VFA was measured using ‘FatScan’, as described previously [15], on one cross-sectional CT image obtained at the level of the umbilicus. Figure 1 shows the method used to determine the fat tissue area on a CT image. First, the intraperitoneal area was defined manually by tracing its contour on the scan (Figure 1a). Thereafter, a region of interest of the subcutaneous fat layer was defined by tracing its contour on each scan, either automatically or manually (Figure 1b), and then the attenuation range of the CT numbers (in Hounsfield units) for the fat tissue was calculated. A histogram for the fat tissue was computed, based on the mean attenuation ± 2 SD (Figure 1c). Within the region outlined in Figure 1a, the tissue with attenuation within the mean ± 2 SD was considered to be the VFA (white arrow, Figure 1d). The total fat area was calculated in the region outlining the circumference of the abdominal wall. The VFA was subtracted and the remainder was regarded as the subcutaneous fat area (black arrow) (Figure 1d).

### Statistical analysis

Differences between groups were examined for statistical significance using the Student’s *t* test with Yates’ correction, the *χ*^2^ test, Fisher’s exact probability test, Wilcoxon rank sum test, or the Mann-Whitney *U* test. Univariate analysis was performed to identify the factors that determined visceral fat loss. The identified variables were subsequently entered into a multivariate analysis using logistic regression analysis to identify independent factors that influenced the visceral fat loss. Significance was assumed for *P* < 0.05. Statistical analysis was performed using *JMP* version 9.0 (SAS Institute Inc, Cary, NC).

## Results

### Comparison of patients’ characteristics according to surgical procedure

Table 1 compares the background characteristics of patients who underwent DGBI and DGRY. Age, sex, body mass index (BMI), and VFA before surgery were not different among the groups. At baseline, the serum albumin concentration was significantly higher in the DGBI (4.25 ± 0.36 mg/dl) than in the DGRY group (4.11 ± 0.36 mg/dl, *P* = 0.0317).

**Table 1:** Comparison of clinical background data of patients who underwent distal gastrectomy-Billroth I and distal gastrectomy-Roux-en-Y

	**DGBI *n *= 104**	**DGRY *n *= 48**	***P***
Age (years)	64 (38 to 85)	62.5 (37 to 80)	0.2574^c^
Sex (M/F)	69/35	37/11	0.1804^a^
BMI (kg/m^2^) before surgery	22.8 (16.0 to 30.5)	22.4 (17.1 to 31.0)	0.7243^c^
VFA before surgery (cm^2^)	75.2 (15.9 to 265.4)	80.6 (14.6 to 236.7)	0.9148^c^
SFA before surgery (cm^2^)	122.5 (35.5 to 267.3)	113.8 (14.9 to 231.5)	0.4142^c^
Albumin level before surgery (mg/dl)	4.3 (2.5 to 4.8)	4.1 (2.8 to 4.7)	0.0128^c^
Approach (laparoscopy/laparotomy)	15/89	0/48	0.0030^b^
Lymph node dissection (<D2/≥D2)	59/45	20/28	0.0840^a^
Omentectomy (yes/no)	13/91	15/33	0.0056^a^
Pathological T (1/2/3/4)	74/28/2/0	32/12/4/0	0.4420^d^
Pathological N (0/1/2/3)	78/20/6/0	34/10/3/1	0.5484^d^
Pathological stage (1/2/3/4)	86/11/7/0	37/5/5/1	0.3587^d^
Adjuvant chemotherapy (yes/no)	10/94	5/43	1.0000^b^
Recurrence (yes/no)	4/100	3/45	0.5110^b^

^a^*χ*^2^ test; ^b^Fisher’s exact test; ^c^Wilcoxon rank sum test; ^d^Mann-Whitney *U* test. *BMI* body mass index, *DGBI* distal gastrectomy with Billroth I, *DGRY* distal gastrectomy with Roux-en-Y, *SFA* subcutaneous fat area, *VFA* visceral fat area.

With regard to the surgical factors, there was a significant difference in surgical approach (that is, the proportion of patients who underwent laparoscopy versus laparotomy) between the DGBI and DGRY groups (*P* = 0.0030), and in omentectomy between the DGBI and DGRY groups (*P* = 0.0056).

Analysis of the pathological factors and postoperative clinical course showed that there was no significant difference between the groups in pathological T, pathological N, pathological stage, adjuvant chemotherapy, or recurrence.

### Comparison of BMI and VFA according to surgical procedure

Table 2 lists comparative data related to BMI and VFA: BMI after surgery and the rate of reduction of BMI (ΔBMI%) were not different between the DGBI and DGRY groups.

**Table 2:** Comparison of indexes of adiposity among patients with distal gastrectomy-Billroth I and distal gastrectomy-Roux-en-Y

	**DGBI *n *= 104**	**DGRY *n *= 48**	***P***
BMI after surgery	20.4 (15.0 to 28.1)	19.7 (16.9 to 26.2)	0.3789^a^
VFA after surgery (cm^2^)	50.8 (6.4 to 167.1)	37.4 (11.7 to 144.5)	0.0742^a^
SFA after surgery (cm^2^)	81.14 (19 to 230.8)	69.9 (23.5 to 184)	0.0808^a^
Alb after surgery (mg/dl)	4.3 (2.4 to 4.7)	4.2 (3.7 to 4.6)	0.3065^a^
Reduction rate of BMI (%)	8.7 (−15 to 31.2)	10.8 (−2.7 to 22.5)	0.1678^a^
Reduction rate of VFA (%)	30.6 (−130.2 to 90.1)	44.2 (−14.5 to 85.2)	0.0027^a^
Reduction rate of SFA (%)	33.0 (−124.6 to 82.3)	31.8 (−147.7 to 87.4)	0.5722^a^

^a^Wilcoxon rank sum test. *BMI* body mass index, *DGBI* distal gastrectomy with Billroth I, *DGRY* distal gastrectomy with Roux-en-Y, *SFA* subcutaneous fat area, *VFA* visceral fat area.

The VFA after DGBI (62.4 ± 39.8 cm^2^) was lower than that after DGRY (50.7 ± 36.2 cm^2^), but the difference was not statistically significant (*P* = 0.0742).

The rate of reduction of the VFA (ΔVFA%) in the DGBI group (24.7 ± 36.8%) was significantly lower than in the DGRY group (42.2 ± 24.1%, *P* = 0.0027). We also examined ΔVFA% in pathological stage I patients. The ΔVFA% in the DGBI group (21.7 ± 38.1%) was significantly lower than in the DGRY group (39.0 ± 24.4%, *P* = 0.0134).

### Determinants of postoperative visceral fat loss

We examined the factors involved in visceral fat loss. For this purpose, the study population was divided into a ‘high VFA loss group’ and a ‘low VFA loss group’ using the value of the median ΔVFA%, which was 36.1%. As shown in Table 3, among the clinicopathological factors examined, adjuvant chemotherapy (yes vs. no, *P* = 0.0006), type of reconstruction (BI vs. RY, *P* = 0146), field of lymph node dissection (<D2 vs*.* ≥D2, *P* = 0.0020), omentectomy (yes vs*.* no, *P* = 0.0003), and pathological stage (1/2 vs. 3/4; *P* = 0.0023) correlated significantly with postoperative visceral fat loss. Multivariate logistic regression analysis that included these factors identified reconstruction (BI vs. RY; *P* = 0.0232) and adjuvant chemotherapy (yes vs*.* no, *P* = 0.0330) as the significant determinants of visceral fat loss (Table 4).

**Table 3:** Results of univariate analysis for visceral fat loss after gastrectomy

**Univariate analysis**	**High VFA loss group**	**Low VFA loss group**	***P***
Sex (M/F)	53/23	53/23	1.0000^a^
Adjuvant chemotherapy (yes/no)	14/62	1/75	0.0006^b^
Reconstruction (BI/RY)	45/31	59/17	0.0146^a^
Lymph node dissection (<D2/≥D2)	30/46	49/27	0.0020^a^
Pathological stage (1 to 2 / 3 to 4)	64/12	75/1	0.0023^b^
Omentectomy (yes/no)	23/53	5/71	0.0003^b^
Surgical approach (laparoscopy/laparotomy)	4/72	11/65	0.1002^b^

^a^*χ*^2^ test; ^b^Fisher’s exact test; *BI* Billroth I, *RY* Roux-en-Y, VFA, visceral fat area.

**Table 4:** Results of multivariate analysis for visceral fat loss after gastrectomy

**Multivariate analysis**	**Hazard ratio**	**95% confidence interval**	***P***
Adjuvant chemotherapy (yes/no)	5.0290	1.1347 to 28.3460	0.0330
Reconstruction (BI/RY)	0.4249	0.1965 to 0.8909	0.0232
Lymph node dissection (<D2/≥D2)	0.9539	0.4482 to 2.0343	0.9022
Pathological stage (1 to 2/3 to 4)	0.4142	0.0723 to 2.0248	0.2774
Omentectomy (yes/no)	0.4862	0.1364 to 1.5993	0.2387

*BI* Billroth I, *RY* Roux-en-Y.

## Discussion

It is reported that body weight loss is mainly caused by loss of body fat [12]. However, the effects of gastrectomy procedures on postoperative visceral fat have not been thoroughly examined. In this study, we analyzed the effect of surgical procedure on VFA. In the early postoperative period, fat volumes are seriously affected by the metabolic abnormality caused by surgery. Previous studies indicated that body weight loss occurs mainly during the first 3 months after surgery [11]. Therefore, in this study, we compared CT images taken at least 6 months after surgery, when the nutritional status is presumed to be stable [16].

A few studies examined changes in total fat or visceral fat after gastrectomy. Liedman *et al*. [12] studied changes in body composition by measuring total body potassium and water in 75 patients with gastric cancer. They observed a significant decrease in total body fat at 6 months after surgery but a subsequent marginal recovery at 12 months. In another study, Kiyama *et al*. [13] evaluated fat mass by multifrequency bioelectrical impedance analysis before and after gastrectomy. They found a larger decrease in body fat mass after TG than after subtotal gastrectomy and laparoscopy-assisted gastrectomy. Yoon *et al*. [17] reported a greater loss of visceral fat tissue after TG than after subtotal gastrectomy at 6 months after surgery. At 12 months after surgery, the losses in BMI, total adipose tissue, subcutaneous, and visceral fat tissues were all greater after TG than after subtotal gastrectomy. Miyato *et al.*[16] reported that postoperative visceral fat of the RY group tended to be smaller than that of the BI group, although the difference was not statistically significant, perhaps because of the small sample size. Based on these differences, there is a possibility that the reconstruction procedure might have some effects on VFA.

The reasons for the differences in visceral fat reduction between BI and RY could not be elucidated in this retrospective study. Previous studies on bariatric surgery are of some help. It is reported that visceral fat reduction was greater after RY gastric bypass than after vertical banded gastroplasty [18]. Although the mechanisms of fat reduction or improvement in insulin resistance are not completely understood in bariatric surgery, the importance of gut hormones has been reported. Among the various gut hormones, gastric inhibitory polypeptide (GIP) and glucagon-like peptide-1 (GLP-1) are reported to regulate fat metabolism. GIP is released from the duodenal endocrine K cells immediately after the absorption of fat or glucose [19]. Furthermore, fat intake induces hypersecretion of GIP, which increases nutrient uptake and triglyceride accumulation in adipocytes [20]. In the context of this study, Korner *et al*. [21] reported lower GIP levels after RY gastric bypass than after adjustable gastric banding, and concluded that the blunted GIP secretion after RY seems to contribute to the greater weight loss and improved glucose homeostasis compared with adjustable gastric banding. GLP-1 is a naturally occurring incretin hormone with a potent blood-glucose lowering action only during hyperglycemia; a GLP-1 analogue reduces visceral fat [22]. Peterli *et al*. [23] reported higher GLP-1 levels after RY gastric bypass than after sleeve gastrectomy. The GIP level might also be lower and the GLP-1 level might be higher after RY than BI, and fat accumulation is lower after RY than BI. Fat malabsorption might be another reason; since clinical tests after RY showed significantly lower fat absorption than after BI and double-tract reconstruction, which accommodated for the passage of food through the duodenum [24].

As for omentectomy, our data showed that it was not a significant determinant of visceral fat loss by multivariate analysis. There are some reports of surgical removal of visceral fat thorough omentectomy [25,26]. There was no significant difference between RY gastric bypass without omentectomy and with omentectomy in fat mass 12 months after surgery [25]; this is compatible with our result.

These results might be helpful in selecting the reconstruction method used after distal gastrectomy, especially for obese patients when remnant stomachs are large enough so that either BI or RY could be performed. However, our study has several limitations. First, a retrospective sample was used in this analysis, and thus the clinical background, such as extent of lymph node dissection, omentectomy, and surgical approach, is different between compared groups, although these factors did not correlate with visceral fat loss in this study. Next, the size of remnant stomach, which could affect the amount of food intake after surgery, might be smaller after RY than after BI, because the former construction technique was often selected when the remnant stomach was too small to allow for anastomosis with the duodenum. Third, the long-term results are unknown, because we examined CT data only 1 year after surgery.

Further prospective studies with stratified randomization and long-term follow-up data, such as VFA after 3 years or 5 years and cause of death, are needed to confirm the effects of reconstruction after gastrectomy on visceral fat.

## Conclusions

Visceral fat loss after RY was larger than that after BI. Further prospective studies are needed to confirm the effects of reconstruction after distal gastrectomy on visceral fat.

## Abbreviations

BI: Billroth I; BMI: Body mass index; CT: Computed tomography; DGBI: Distal gastrectomy with Billroth I; DGRY: Distal gastrectomy with Roux-en-Y; GIP: Gastric inhibitory polypeptide; GLP-1: Glucagon-like peptide-1; RY: Roux-en-Y; SD: Standard deviation; VFA: Visceral fat area.

## Competing interests

The authors declare that they have no competing interests.

## Authors’ contributions

KT collected data, performed analysis and drafted the manuscript. IM conceived this study, helped to draft the manuscript and participated in the treatment of these patients. MY participated in study design, literature search, and coordination. OI participated in study design and helped to draft the manuscript. KK, MM, TS, SN, MO, and HO participated in the treatment of these patients. All authors read and approve the final manuscript.
